# First report on outbreaks of contagious ovine digital dermatitis in Sweden

**DOI:** 10.1186/s13028-021-00595-x

**Published:** 2021-08-16

**Authors:** Malin Bernhard, Sara Frosth, Ulrika König

**Affiliations:** 1 Farm & Animal Health, Kungsängens Gård, 753 23 Uppsala, Sweden; 2 Department of Biomedical Sciences and Veterinary Public Health, Swedish University of Agricultural Sciences, PO. Box 7036, SE-750 07 Uppsala, Sweden

**Keywords:** Footrot, *Treponema*, Lameness, Sheep, *Dichelobacter nodosus*, *Fusobacterium necrophorum*

## Abstract

**Background:**

Contagious ovine digital dermatitis (CODD) is considered widespread in the United Kingdom but was only recently reported in mainland Europe, as one outbreak in Germany. The disease can cause severe lameness in sheep and, if left untreated, can lead to total avulsion of the hoof capsule. CODD is considered to have multifactorial and polymicrobial aetiology, in which *Treponema medium/Treponema vincentii* phylogroup, *Treponema phagedenis* phylogroup and *Treponema pedis* are believed to play a significant role. Footrot and CODD have a close connection and footrot is considered an important risk factor for CODD.

**Case:**

Lameness, mainly in lambs aged 1.5 months, was reported on a farm in Sweden in spring 2018. The animals showed no signs of footrot and the causative agent, *Dichelobacter nodosus*, was not found. CODD was suspected but not confirmed, and the clinical signs subsided when the animals were turned out to pasture. In February 2019, young lambs and ewes were lame again and this time CODD was diagnosed. After treatment, the whole flock was slaughtered later in 2019 due to CODD.

In autumn 2020, CODD was diagnosed on another Swedish farm, this time as part of a mixed infection with *D. nodosus.* The animals were treated with footbaths in zinc sulphate 10% by the farmer, but lameness recurred soon afterwards. The animals were treated, but ultimately the whole flock was slaughtered. No connection was found between the two farms.

**Conclusion:**

The first two outbreaks of CODD in Sweden have been diagnosed and are described in this case report. If it spreads, CODD could have a negative impact on the Swedish sheep industry in terms of animal welfare, production and antibiotic use.

## Background

Contagious ovine digital dermatitis (CODD) was first described in 1997 in the United Kingdom [1], where the disease is now considered endemic and widespread, with 35–58% of sheep farmers reporting occurrence of the disease in their flocks [2–4]. CODD has also been reported in Ireland [5] and the first confirmed outbreak in mainland Europe occurred in Germany in 2018 [6]. According to recent surveys, within farm-prevalence of CODD in the United Kingdom is approximately 2% [2–4] but can be 20–50% on individual farms [2, 3, 7, 8]. There is a seasonal pattern, with a small rise in clinical cases in spring and a higher peak in late summer/early autumn [9].

Diagnosis of CODD is based on clinical findings. The lesions can be graded from 1–5, describing how the disease progresses over time and how much of the hoof capsule is involved [10] (Table 1). Early cases typically present with erosion/ulceration at the coronary band, with or without lameness. As the lesion progresses, the hoof capsule becomes separated from the underlying tissue, causing severe lameness, and possibly resulting in total avulsion of the hoof capsule. As the lesion heals, both the horn and underlying bone can become deformed, potentially causing chronic lameness [10].

**Table 1:** Grading system for CODD according to Angell *et al*. [10]

Grade	Description
1	Focal lesion, either proliferative or erosive/ulcerative, affecting the digital skin and coronary band. No underrunning of the hoof capsule
2	Separation between the hoof capsule and the lamellae affecting < 50 % of the hoof wall. Swollen digits. Subcorneal tissue haemorrhagic often with purulent material adherent. Foul smell
3	As grade 2 but affecting 50–100 % of the hoof wall
4	Horn beginning to regrow. Exposed subcorneal tissue roughened, haemorrhagic/necrotic, friable and easily traumatised. Affected digits often distinctly swollen and shortened
5	Horn regrown, surface smooth but distorted by circumferential ridges or creases. Digit often still wide and short

The aetiology of CODD is still unclear but is considered multifactorial and polymicrobial [9, 11, 12]. Strong association to spirochetes [12, 13] and more specifically *Treponema medium/Treponema vincentii* phylogroup, *Treponema phagedenis* phylogroup and *Treponema pedis*, has been observed [5, 14]. These same treponeme phylogroups are considered to cause bovine digital dermatitis (BDD) [15]. *Dichelobacter nodosus* and *Fusobacterium necrophorum* have also been found in CODD lesions [14] and clinical footrot caused by *D. nodosus* is a risk factor for CODD according to several studies [4, 9, 16]. It has recently been suggested that footrot and CODD should be regarded as different stages of the same ovine infectious foot disease [12]. Other potential risk factors identified include sheep being housed, adult sheep compared to lambs, cattle with BDD on the farm [3], large sheep flocks [2, 3, 9], lush pasture [9] and poor biosecurity practices [2].

CODD does not respond to conventional footrot treatment with footbathing in formalin or zinc sulphate solution [1, 6]. *In vitro* studies have shown that *Treponema* spp. isolated from CODD are sensitive to several antibiotics, including penicillin [17]. Whole-flock injection with long-acting amoxicillin, in combination with footbathing in chlortetracycline, has been shown to increase the probability of recovery and to lower the rate of new infections, compared with footbathing alone [7]. A single dose with long-acting amoxicillin administered to affected animals has been shown to have a 71% cure rate after 9 weeks [16]. However, it is unclear what the cure rate would have been without amoxicillin. In an experimental study, two injections with amoxicillin 48 h apart cured all lesions within 7 days [12]. In a pilot study, two injections with tilmicosin, 2 weeks apart, achieved a 100% cure rate 4 and 6 weeks after the first treatment [18]. That pilot study was followed by an attempt to eradicate CODD on flock level through whole-flock treatment with tilmicosin and isolation of affected animals. Affected animals were given a second dose of tilmicosin 2 weeks after the first injection. The protocol used did not show a significantly higher rate of eradication in treated flocks, so the use of whole-flock metaphylactic treatment with tilmicosin is inadvisable considering the importance of tilmicosin in human medicine. In the German case, lame sheep were successfully treated individually with gamithromycin [6].

There were 279,888 ewes and rams on 8463 registered holdings in Sweden 2019, resulting in an average flock size of 33 ewes [19]. Footrot was first diagnosed in Sweden in 2004 [20]. The diagnosis is based on lesions with a score ≥ 2, according to the 5-point scoring system developed by Stewart and Claxton [21] (Table 2). Since 2009, there has been a voluntary control programme against footrot in Sweden [22]. The programme aims to eliminate the disease from affected farms and to enable trading with certified animals from flocks without footrot. The prevalence of footrot has since decreased significantly, according to prevalence studies of Swedish slaughter lambs [23, 24]. Out of randomly selected lambs at abattoirs in 2009 and 2020, 5.8% [23] and 1.8% [24], respectively, showed clinical signs of footrot (score ≥ 2). During the same period, new Swedish cases of footrot, reported within the control programme, decreased from around 20–30 to ≤ 5 cases annually [22]. In 2019, CODD was diagnosed for the first time in Sweden, followed by a second outbreak in 2020. The aim of this report is to describe these two outbreaks.

**Table 2:** Scoring system for footrot according to Stewart and Claxton [21]

Score	Description
1	Inflammation to the interdigital skin with erosion of the epithelium
2	Necrotising inflammation of the interdigital skin and part or all of the soft horn of the axial wall
3	Necrotising inflammation and underrunning of the soft horn of the heel and sole, not extending to the abaxial edge of the sole
4	Underrunning extending to the abaxial edge of the sole
5	Necrotising inflammation of the laminae of the abaxial wall and underrunning of the hard horn

## Case presentations

### Case presentation 1

Farm 1, which is situated in Skåne county in southern Sweden, started keeping sheep in 2015. Both ewes and rams were purchased from several different farms in Sweden. In 2018, the flock consisted of 40 Gotland Pelt ewes and 100 crossbreed ewes.

In April 2018, Farm & Animal Health, an advisory company working with animal health issues in the sheep, pig and cattle industries and runs control programmes approved by the Swedish Board of Agriculture in cooperation with The National Veterinary Institute, was contacted by the farmer regarding lameness. A local veterinary surgeon had visited the farm and prescribed benzylpenicillin (Penovet® vet 300 mg/mL, Boehringer Ingelheim Animal Health) and meloxicam (Metacam® 20 mg/mL, Boehringer Ingelheim Animal Health) for the lame sheep. Since footrot was suspected, the farm was enrolled in the national footrot control programme and the flock was examined by a veterinarian from Farm & Animal Health. Several lame lambs aged 1.5 months, mainly of the Gotland Pelt breed, were observed. At clinical examination, the medio-dorsal hoof horn at the coronary band appeared dissolved, while the interdigital skin, sole and heel were not affected. CODD was suspected, although the disease had not been diagnosed in Sweden previously, but because of the non-specific clinical signs the diagnosis could not be confirmed.

Five swabs taken from the interdigital space and from the lesions at the coronary band were analysed for the presence of *D. nodosus*, *F. necrophorum* and *Treponema* spp., by real-time polymerase chain reaction (PCR) as previously described [25]. One swab tested positive for *F. necrophorum* subsp. *funduliforme*, but all other swabs tested negative in the three different real-time PCR methods. In addition, an attempt was made to culture *Treponema* spp., as described in Svartström et al. [26], but this was unsuccessful.

Two weeks later, the farmer reported that there had been no effect of the treatment with penicillin and that lameness in the flock had progressed, with more lame lambs. As BDD can be successfully treated with salicylic acid [27], some of the lambs had salicylic acid vaseline mixed with salicylic acid powder applied to the lesions. This treatment also had no effect. The treatment was then changed to topical administration of chlortetracycline hydrochloride spray (Cyclospray vet, Eurovet Animal Health BV), which had a good effect on affected animals. This treatment was repeated for the whole flock in May, just before turnout to pasture. During pasture and later during autumn and early winter, no lameness was observed in the sheep, but growth rate of the hoof horn was abnormally fast. According to the farmer, the feet looked like they had not been trimmed for many years. Both the hard horn, the soft horn of the heel and sole were overgrown and appeared softer than usual. Some of the ewes with overgrown feet had low body condition scores.

**Fig. 1:**
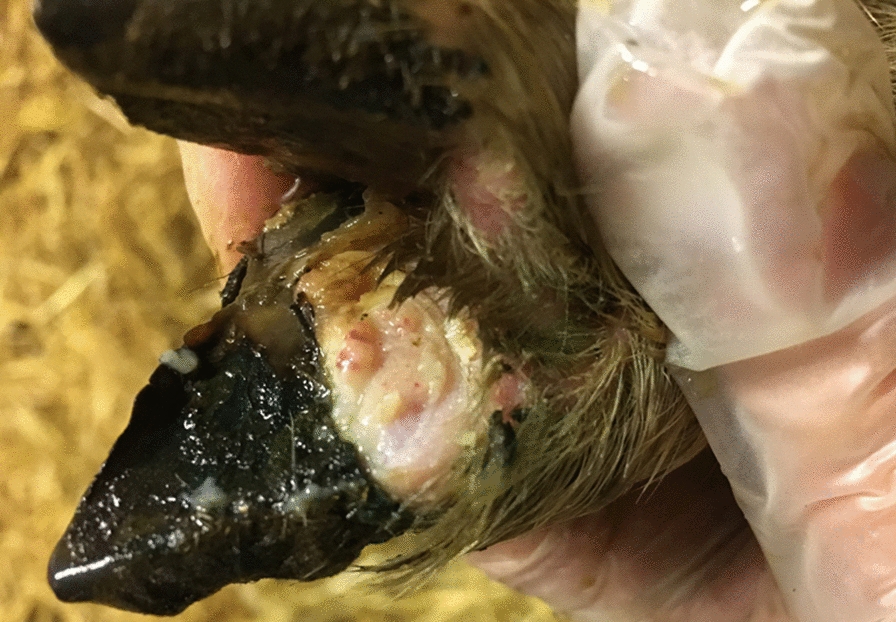
Proliferative lesion (Grade 1) in the medial part of the coronary band of a two-month-old lamb on Farm 1. Photo: Johanna Haraldsson

In February 2019, lambs aged 1.5–2 months and some ewes in the group of crossbreeds showed signs of lameness. At clinical inspection, there were no signs of footrot score 2–5. However, in the medio-dorsal part of the coronary band there were proliferative lesions (Fig. 1), which were much more evident than in the previous year. In some lambs, the hoof capsule distal to the medio-dorsal part of the coronary band had separated from the underlying tissue. Treatment with chlortetracycline hydrochloride spray had a good initial effect, but relapse occurred soon after treatment. Photos were sent to Jennifer Duncan BVM & S BSc. (Hons) PhD Dip. ECSRHM Dip. VEPH MRCVS, University of Liverpool, who confirmed that the clinical picture of the lesions together with the case history were indicative of CODD.

Five additional swabs taken from the interdigital space and from the lesions at the coronary band were again analysed for presence of *D. nodosus*, *F. necrophorum* and *Treponema* spp., in the same way as in 2018. Again, all swabs tested negative for *D. nodosus*, but two swabs tested positive for *F. necrophorum* subsp. *funduliforme* and all swabs tested positive for *Treponema* spp. in real-time PCR. Attempt to culture *Treponema* isolates were again unsuccessful.

As more sheep became lame and relapses occurred after treatment, the chlortetracycline spray was complemented with injections of oxytetracycline (Vetroxy vet 200 mg/mL, Bimeda Animal Health Limited) and meloxicam (Metacam® 20 mg/mL, Boehringer Ingelheim Animal Health). About one month after the first signs of lameness, approximately 40% of the lambs were treated, but only a few ewes. Several relapses occurred and proliferative lesions at the coronary band were also observed in lambs without lameness. Gamithromycin (Zactran® 150 mg/mL, Boehringer Ingelheim Animal Health) was then administered to all crossbreed ewes and to young lambs. Due to the long withdrawal time before slaughter for gamithromycin, the older lambs were kept separately and treated only with oxytetracycline spray. As in the previous year, the hoof horn of all animals showed abnormally high growth rate during both the housing and pasture periods.

Because CODD is a new disease in Sweden, the outbreak was reported to the authorities as the first index case. The sheep farmers’ organisations worked together with the authorities and Farm & Animal Health to distribute information about CODD to Swedish sheep farmers. To prevent national spread of the disease, with negative consequences for animal welfare, production and antibiotic use, the Swedish Board of Agriculture decided to compensate the sheep farmer for stamping out the flock. In July 2019, all sheep were slaughtered.

### Case presentation 2

The flock on Farm 2, also located in Skåne County, consisted of 25 ewes, five adult rams and 27 lambs. Twenty of the ewes and their lambs born in February–April were bought into the farm in April 2020. The bought-in sheep were kept in a separate pen during the housing period. Some weeks after turnout to pasture, lameness was observed among lambs in the flock, most frequently among lambs born to the original five ewes, which had no history of lameness.

Examinations performed by the sheep farmer revealed footrot score 2–3 in lame sheep, but no lesions in the coronary band were observed. The farmer estimated that 5% of the sheep were lame at this point. The flock was treated with footbath in zinc sulfphate 10% on three occasions between the end of July and first week of September. After every footbath, the flock was moved to a clean surface indoors where no sheep had been for the previous 3 weeks. After the last footbath, the animals were turned out to a pasture that no sheep had grazed for the previous 3 weeks. The treatment was initially effective, but 2 weeks later lameness was again observed in the flock.

**Fig. 2:**
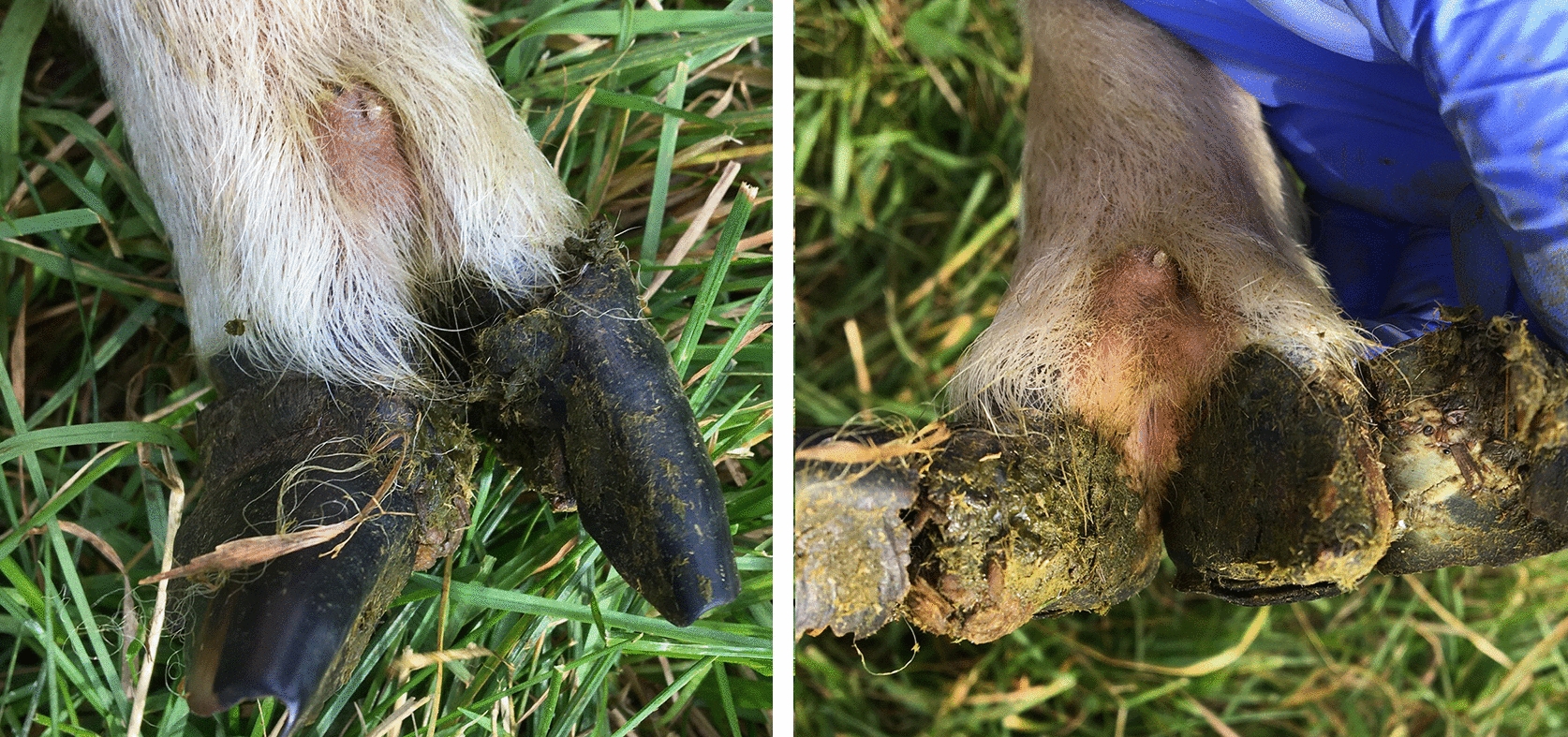
CODD lesion grade 5 on left hind foot of ram lamb number 12 on Farm 2 before and after the loose horn was pulled away from the hoof. Photo Emelie Larsdotter

**Fig. 3:**
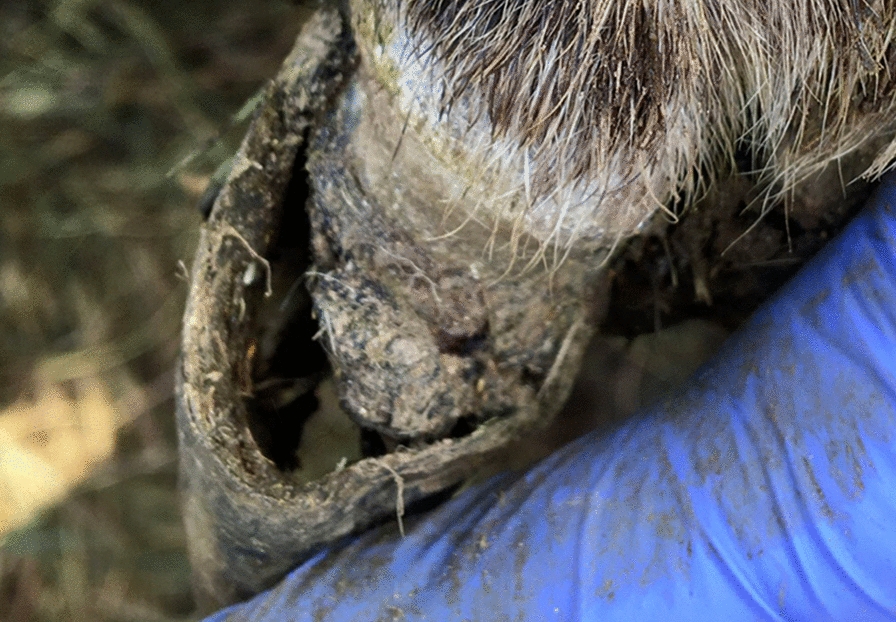
Right hind foot of ewe number 7 on Farm 2, loose horn and the horn covering the healed CODD-lesion (grade 5) had a dried appearance. Photo Emelie Larsdotter

**Fig. 4:**
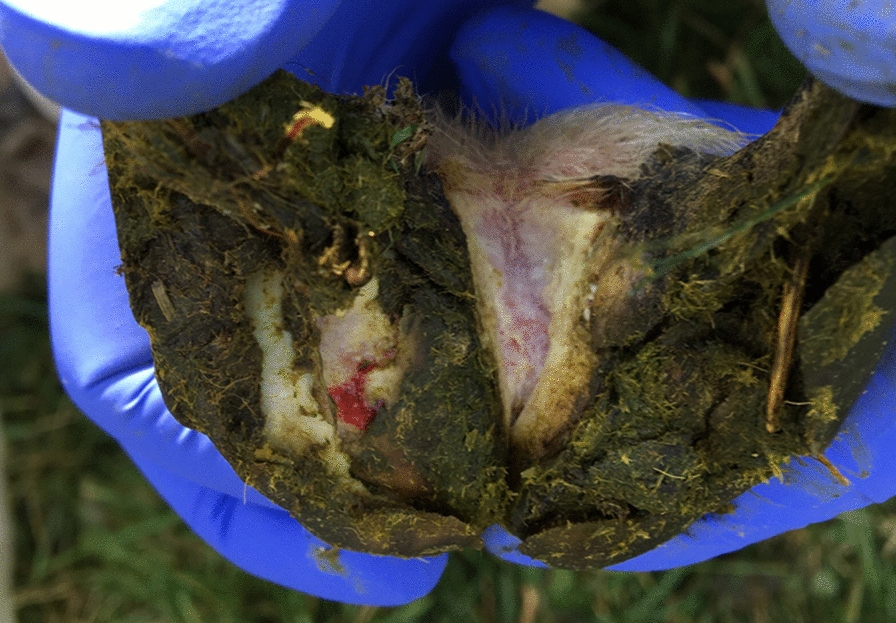
Left hind foot of ram lamb number 11 on Farm 2 with footrot score 3. Photo Emelie Larsdotter

At the end of September 2020, Farm & Animal Health was contacted by the farmer regarding the lameness in the flock. By that time, one lamb had a wound at the coronary band and a detached hoof capsule. The flock was enrolled in the national footrot control program and was examined in early October by veterinarians from Farm & Animal Health. In total, all 25 ewes, the five rams and 13 ram lambs were examined. The 14 ewe lambs belonging to the flock were not on the farm during the visit and no lameness had been observed among them at that point. Both footrot score 2–4 and CODD lesions grade 1–2 and 4–5 were observed (Tables 3, 4, Figs. 2, 3, 4). Photos of the lesions were again sent to Jennifer Duncan BVM & S BSc. (Hons) PhD Dip. ECSRHM Dip. VEPH MRCVS and Joseph Angell BVSc MSc Dip. LSHTM PhD MRCVS, University of Liverpool, and they confirmed that the clinical picture indicated CODD. In most of the CODD lesions, the hoof horn capsule was detached from the underlying tissue and in most cases the avulsion seemed to have begun from the axial groove and proceeded vertically along the dorsal aspect of the hoof horn (Fig. 2). At the most lateral part of the hoof, the capsule was still attached in some cases (Fig. 2).

**Table 3:** Results of clinical examination (performed by veterinarians in October 2020) of sheep in the second Swedish case (Farm 2) of contagious ovine digital dermatitis (CODD): prevalence of footrot lesions and CODD

	Number of sheep	Footrot score 2–5 (%)	CODD (%)	Footrot score 2–5 and CODD on the same foot (%)
Ewes	25	8 (32)	4 (16)	3 (12)
Rams	5	3 (60)	1 (20)	1 (20)
Ram lambs	13	5 (38)	2 (15)	2 (15)
Total	43	16 (37)	7 (16)	6 (14)

**Table 4:** Clinical findings of footrot/contagious ovine digital dermatitis (CODD) and bacteriological results from hoof swabs from Farm 2

ID	Adult/ lamb	Sex	Footrot score/CODD grade				*Treponema* spp. PCR	Treponema BDD phylogroups PCR	Treponema culture	*Dichelo-bacter nodosus* 16S PCR	*D. nodosus* vir PCR	*D. nodosus* culture, conf. MALDI-TOF and *D. nodosus* vir PCR	*Fusobacterium necrophorum* subsp. *necrophorum* /*funduliforme* PCR
			Fore-leg left	Fore-leg right	Hind-leg left	Hind-leg right							
1	Adult	Ewe	1/2	2/5	0/0	0/0	n.a.	n.a.	n.a.	n.a.	n.a.	n.a.	n.a.
2	Adult	Ewe	1/0	2/0	0/0	0/0	+	−	−	+	+	+/+/+	+/−
3	Adult	Ewe	3/0	3/0	0/0	0/0	n.a.	n.a.	n.a.	n.a.	n.a.	n.a.	n.a.
4	Adult	Ewe	0/0	0/0	0/4	0/0	n.a.	n.a.	n.a.	n.a.	n.a.	n.a.	n.a.
5	Adult	Ewe	4/0	0/0	0/0	0/0	+	−	−	+	+	n.a.	+/+
6	Adult	Ewe	0/0	3/0	0/0	0/0	+	−	−	+	+	+/+/+	+/−
7	Adult	Ewe	0/0	0/0	2/0	5/5	+	−	−	+	+	n.a.	+/+
8	Adult	Ram	2/0	2/0	0/0	3/0	+	−	−	+	+	n.a.	+/−
9	Adult	Ram	0/0	0/0	3/1	0/0	+	−	−	+	+	n.a.	+/−
10	Lamb	Ram	0/0	0/0	2/4	0/0	+	−	−	+	+	+/+/+	+/+
11	Lamb	Ram	0/0	0/0	3/0	0/0	n.a.	n.a.	n.a.	n.a.	n.a.	n.a.	n.a.
12	Lamb	Ram	0/0	0/0	2/5	0/0	+	−	−	+	+	n.a.	+/−

*+ *positive, *−* negative, *n.a* not analysed

Eight swabs taken from the interdigital space and from the lesions at the coronary band or from the areas of detached hoof horn were analysed for the presence of *D. nodosus*, *F. necrophorum* and *Treponema* spp. in the same way as for Farm 1. An additional real-time PCR for *Treponema* spp. using a probe-based approach [28] was performed for the swabs from Farm 2 and *Treponema* spp.-positive swabs were also analysed for the three BDD-associated *Treponema* phylogroups [15]. Three swabs were also cultivated for *D. nodosus* [21]. All swabs tested positive for virulent *D. nodosus*, *F. necrophorum* subsp. *necrophorum* and *Treponema* spp., but negative for the BDD-associated *Treponema* phylogroups (Table 4). Three swabs also contained *F. necrophorum* subsp. *funduliforme*. No *Treponema* isolates could be cultivated, but the three swabs cultivated for *D. nodosus* were all positive, findings confirmed by matrix-assisted laser desorption/ionization time-of-flight mass spectrometry (MALDI-TOF MS) (Bruker Daltonics, Billerica, MA, USA). Whole-genome sequencing of the three *D. nodosus* isolates, performed as described in Hansson et al. [29], revealed that these isolates were of sequence type (ST) 1 and serogroup G, and were virulent.

The farmer and veterinarians together made the decision to treat the flock so that all sheep could be sent to slaughter as soon as possible. Seven sheep were culled due to extensive CODD, severe footrot, other cause of lameness and/or poor growth. Five sheep with CODD lesions received two injections of amoxicillin (Vetrimoxin vet 150 mg/mL, Ceva Animal Health), with one day between injections. The whole flock was housed and every weekend until slaughter (November 20), the whole flock was examined by the farmer and all sheep were footbathed. During the period from October to the slaughter date, 50% of the lambs and 20% of the adult sheep were showing clinical signs of footrot score 2 on at least one occasion. These individuals were treated with chlortetracycline hydrohloride spray on the day after footbathing when they showed clinical signs.

No more signs of CODD grade 1–4 or footrot score 3–5 were observed by the farmer. After slaughter, one foot on an ewe lamb showed signs of CODD grade 5.

## Discussion and conclusions

The clinical picture differed considerably between the two farms and the distribution between different grades of CODD was different from that previously reported [9]. In the first outbreak (Farm 1), the animals were housed and the farmer contacted Farm & Animal Health immediately upon discovering lameness. That resulted in only CODD grade 1–3 being seen on visits and lameness was marked in most affected animals. In the second case (Farm 2), the lameness problem had started months before the clinical examination, resulting in CODD grade 4–5 being present. The fact that the animals had been footbathed in zinc sulphate might have lowered the incidence, explaining why only one animal was graded with CODD 1, one with CODD 2 and none with CODD 3. In the second outbreak animals with CODD lesions did not all show clinical signs of lameness, which could be expected as the probability of lameness is lower for CODD grade 4–5 compared with grade 2–3 [9]. The farmer reported that only around 5% of the animals were lame during the summer, but as many as 37% had footrot and 16% had CODD lesions at clinical examination (Table 3). This indicates how difficult it can be for farmers to discover sheep with foot diseases only by looking for lameness. Close inspection of the feet, not only of lame sheep, is necessary for an overall interpretation of the situation in the flock as not all sheep with CODD show lameness [9].

At the first farm (April 2018), the clinical cases subsided when the animals were moved out to pasture and no more cases were seen until February 2019. One reason why no rise in clinical cases was seen even during late summer and early autumn [9] could be the extremely dry summer of 2018. Another explanation could be the very low stocking density at pasture compared with when the sheep were housed with their lambs.

In the United Kingdom, CODD is reported to have a higher incidence in ewes than in lambs [8], but in the first Swedish outbreak most affected animals were lambs, some as young as 1.5 months. The reason for the different age distribution of CODD might be that in UK the lambs go out to pasture and a low-risk environment sooner after birth, so there is not enough time for the disease to develop during that risk period [9].

In the first outbreak, no signs of footrot (score 2–5) were seen when examined by a veterinarian with long experience of working with footrot. Absence of footrot on Farm 1 was confirmed when *D. nodosus* was not found in swabs in either 2018 or 2019. However, the absence of *Treponema* spp. in 2018 turned out to be a false-negative result, as all swabs tested positive when diluted and re-analysed the following year. This was done because the swabs from 2019 tested positive for *Treponema* spp. but showed signs of inhibition. It is clear that the method used to detect *Treponema* spp. [25] was sensitive to inhibition and lacked a control for this. Thus, an additional real-time PCR method, which was probe-based [28] and which included an internal control, was used to detect *Treponema* spp. in the swabs from Farm 2. Although all swabs from the two outbreaks were positive for *Treponema* spp., it is unclear what this means from a diagnostic perspective. None of the three BDD-associated *Treponema* phylogroups were detected in swabs from Farm 2. However, Duncan et al. [12] found that *Treponema* spp. are more often present in samples from deeper tissue than superficial tissue, so the possibility of finding the phylogroups associated with BDD could have been greater if biopsies had been analysed instead of swabs. *Treponema* spp. has previously been found on healthy feet in Swedish sheep [25]. More research is needed regarding the *Treponema* species present on sheep feet in Sweden.

The fact that footrot is less common in Sweden compared with UK suggests that CODD could have less possibilities to become widespread in Sweden. However, the first outbreak in Sweden indicated that *D. nodosus* is not required for the development of CODD. Furthermore, the peak of clinical cases in spring seen in the United Kingdom is thought to occur partly due to housing [3]. In Sweden, the housing period is longer, lasting from November until late April. It is also common for large meat-producing farms to have winter lambing, as was the case on Farm 1, placing young lambs in a potential risk environment for a long period.

In the first case the farmer reported no effect after parental benzylpenicillin treatment even though strains of *Treponema* spp. from CODD have shown to be sensitive for penicillin [17]. Treatment with gamithromycin did have a good effect according to the farmer on Farm 1, when used in the second year. The reasons why gamithromycin had a better effect than benzylpenicillin could be its long persistence after treatment and possibly better distribution to the affected tissue. Topical administration of chlortetracycline had a good initial effect on the CODD 2 lesions in sheep on Farm 1, but were soon followed by relapses. A limited effect of topical chlortetracycline has been observed in the United Kingdom and Germany [6, 7]. On Farm 2, most of the CODD lesions were healed or almost healed (grade 4–5) at the time of the veterinary inspection, but the farmer did not report any failure of treatment with amoxicillin for those animals that received treatment.

The source of the infection was not identified in either of the two outbreaks and there was no known contact between the two farms. Other farms that had contact with Farms 1 and 2 did not report any lameness issues or clinical signs of CODD in their flocks. The fact that CODD was found on two farms with no obvious connection indicates that there may be more unidentified cases in Sweden. In the German outbreak, it was suggested that the infection spread from dairy cattle [6]. In southern Sweden, more than 60% of dairy farms had at least one case of BDD during 2019, while this infection is considered rare in beef cattle (Ann Nyman, MSc PhD Senior Lecturer, Växa Sverige, personal communication 2021). In Sweden, cattle rarely co-graze with sheep, especially not dairy cattle. In the first case, the sheep had been co-grazing with beef cattle, but no clinical BDD cases were reported on that farm. Transmission of *Treponema* spp. from the cattle can however not be excluded. The second outbreak had no known contact with cattle. Another route of transmission could be from imported animals. Neither of the two farms had imported any animals. However, a few animals have been imported to Sweden from different European countries, including the United Kingdom, in the past few years which makes it a possible route of entrance of the infection to Swedish farms.

In both outbreaks our recommendation to the sheep farmers concerned was to prepare the flocks for slaughter as soon as possible. This recommendation was made in consultation with the Swedish sheep farmers’ associations and the authorities to prevent spread of the infection. Sweden currently has the lowest usage of antibiotics for farm animals in the EU [30]. However, if CODD were to become widespread, that could result in a drastic increase in antibiotic use within the Swedish sheep industry, besides threatening animal welfare and lowering production. In Sweden, footrot has been successfully eliminated on flock level and there are several other national programmes aiming to control or eliminate specific infectious diseases in farm animals [22]. In the United Kingdom, attempts to eliminate CODD on flock level with metaphylactic tilmicosin have failed [18], and no other strategy has yet been presented for elimination of CODD in a flock.

On investigating these first two outbreaks of CODD in Sweden, we experienced the importance of international collaboration. When a new disease appears and knowledge is limited nationally, it is essential to take advantage of support from experienced colleagues abroad. As an outbreak of CODD recently has been reported in Germany [6], it is most likely that CODD is an emerging disease in Europe. It is important to be aware of the typical clinical signs of CODD and other differential diagnoses, and to monitor the prevalence worldwide. Finally, we encourage more research, especially studies on elimination of CODD at flock level and on the design of reliable laboratory tests.

In summary, the first two outbreaks of CODD in Sweden have been diagnosed. If it spreads, CODD could have a negative impact on the Swedish sheep industry in terms of animal welfare, production and antibiotic use.

## Data Availability

The datasets used are available from the corresponding author on reasonable request.
